# Deep eutectic-solvothermal synthesis of nanostructured ceria

**DOI:** 10.1038/ncomms14150

**Published:** 2017-01-25

**Authors:** Oliver S. Hammond, Karen J. Edler, Daniel T. Bowron, Laura Torrente-Murciano

**Affiliations:** 1 Centre for Sustainable Chemical Technologies, Department of Chemistry, University of Bath, Bath BA2 7AY, UK; 2 ISIS Neutron and Muon Source, Science and Technology Facilities Council, Rutherford Appleton Laboratory, Didcot OX11 0QX, UK; 3 Department of Chemical Engineering and Biotechnology, University of Cambridge, Pembroke Street, Cambridge CB2 3RA, UK

## Abstract

Ceria is a technologically important material with applications in catalysis, emissions control and solid-oxide fuel cells. Nanostructured ceria becomes profoundly more active due to its enhanced surface area to volume ratio, reactive surface oxygen vacancy concentration and superior oxygen storage capacity. Here we report the synthesis of nanostructured ceria using the green Deep Eutectic Solvent reline, which allows morphology and porosity control in one of the less energy-intensive routes reported to date. Using wide Q-range liquid-phase neutron diffraction, we elucidate the mechanism of reaction at a molecular scale at considerably milder conditions than the conventional hydrothermal synthetic routes. The reline solvent plays the role of a latent supramolecular catalyst where the increase in reaction rate from solvent-driven pre-organization of the reactants is most significant. This fundamental understanding of deep eutectic-solvothermal methodology will enable future developments in low-temperature synthesis of nanostructured ceria, facilitating its large-scale manufacturing using green, economic, non-toxic solvents.

## Abstract (web summary)

Synthesis of nanostructured ceria on green deep eutectic solvent reline is one of the less energy-intensive routes to date. Here, the authors show that the reline solvent plays the role of a latent supramolecular catalyst driving the pre-organization of the reactants, allowing morphology and porosity control.

Cerium (IV) oxide (ceria) is the stable, pale yellow oxide form of the most abundant rare earth metal. Driven by a ground-state cerium 4*f* electron, the powerful Ce^3+^ to Ce^4+^ redox couple^1^, alongside rapid oxygen diffusion facilitated by a cubic fluorite structure, makes ceria a responsive oxygen buffer, with lattice oxygen abstracted or replenished depending on the chemical environment^2^. Ceria is therefore a technologically important material for catalytic oxidation of hydrocarbons and CO, and consequently it has a significant application in automobile emission control, in particular when doped with other transition metals^3^. Approaching the nanoscale, ceria catalysts become profoundly more active due to their enhanced surface area to volume ratio, reactive surface oxygen vacancy concentration and superior oxygen storage capacity^4^. The catalytic activity, especially at low temperatures, can be further enhanced by controlling the morphology at the nanoscale; one-dimensional (1D) assemblies such as nanorods selectively expose the highly reactive (100) and (110) crystal planes, enhancing activity further^5^. True morphological control over nanoceria is therefore a significant milestone, with the potential to negate the requirement for the addition of scarce precious metals to achieve sufficiently active catalysts even at low temperatures. Several synthetic avenues exist for this purpose, which are addressed in a recent review by Sun *et al.*^6^. Hydrothermal conditions are particularly malleable;^7^,^8^,^9^,^10^ for example, various ceria nanostructures are obtained by varying reaction time, temperature or base concentration^11^, surfactant self-assembly can provide additional morphological control^12^ and urea can be introduced to form fractal, dendritic ceria from hydrolysis products^13^. Solvothermal methods, reviewed by Walton^14^, are a particularly interesting development, as synthetic control is obtained by direct modification of the solvent environment. In this context, Deep Eutectic Solvents (DESs) are an extended class of ionic liquids (ILs) made by complexing a (typically ammonium halide) salt with hydrogen bond donor molecules, depressing the glass transition temperature (*T*_g_) at the eutectic molar ratio^15^. Similar to ILs, DESs are green solvents with low vapour pressure and a tunable nature; the hydrophobicity and physicochemical properties of the solvent can be altered by changing the salt or hydrogen bond donor, or by addition of various additive compounds^16^,^17^,^18^. DESs are prepared from many species, including metal ions and plant metabolites. Choline chloride (ChCl) systems have gathered the most interest, with the 1:2 ChCl:urea DES (reline) proving most popular, due to being particularly tractable, low cost, biodegradable and non-cytotoxic^19^,^20^. Primarily, DESs have found use as media for metal electrodeposition^21^, but are also applied in metal-catalysed organic synthesis^22^ and nanomaterial synthesis^23^, alongside separation/extraction applications such as biodiesel purification^24^ and CO_2_ sequestration^25^. Ionothermal synthesis, an IL analogue of hydro/solvothermal conditions, has recently been developed for synthesizing metal-organic frameworks and zeolitic materials using DESs^26^,^27^,^28^26–^28^. In these syntheses, the solvent environment acts as an organic structuring framework, whereas the DES delivers templating agents from thermal degradation.

## Results

### Synthesis

**Figure 1:**
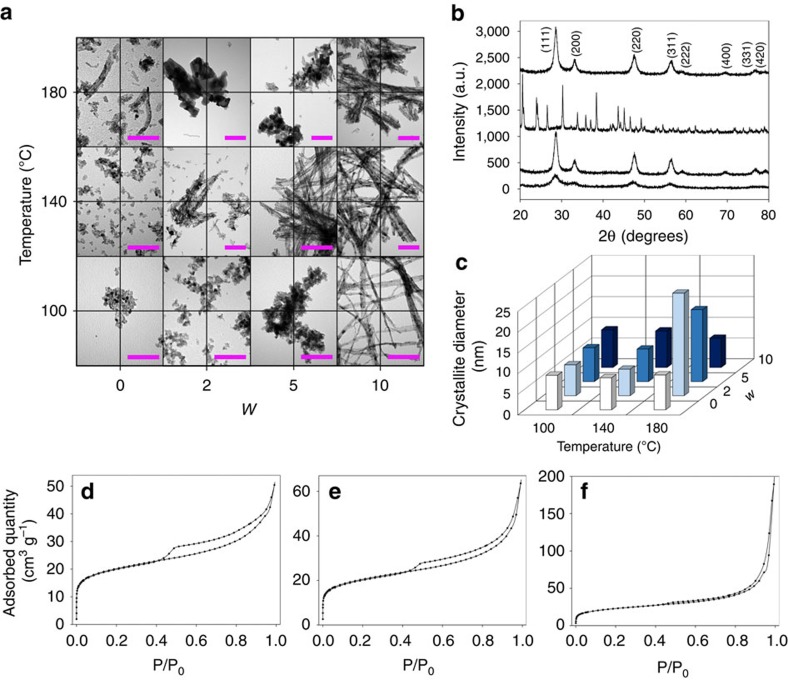
Characterization of deep eutectic-solvothermally synthesized ceria nanostructures. (**a**) Representative TEM images of ceria materials after calcination (scale bars depict 100 nm), showing the effect of synthesis temperature and DES hydration ratio (*w*) on the morphology of the materials; (**b**) XRD patterns of representative ceria materials before and after calcination. (**c**) Mean crystallite size of calcined materials as determined by Scherrer analysis of XRD data. (**d**–**f**) N_2_ adsorption isotherms at 77 K of the Ce-*x*-10 materials, demonstrating the effect of synthetic temperature on the porosity of the materials. Ceria structures are labelled as Ce-*x*-*y*, where *x* is synthesis temperature and *y* the DES molar hydration ratio (*w*).

These previous studies have inspired us to develop a novel solvothermal synthesis protocol using DESs for morphology-tunable nanoceria synthesis. We demonstrate that reline acts as a latent supramolecular catalyst by bringing the reactive components together in the presence of water, which at the same time acts as a directing agent. Reline and its aqueous mixtures are compatible with metal ions, including common ceria precursors such as Ce(NO_3_)_3_·6H_2_O or CeCl_3_, negating the need for the high concentration of solubilizing base that is required in equivalent hydrothermal synthesis. In addition, we use catalytic data to relate the physicochemical properties of the resulting ceria materials with the synthetic conditions. We initially trialed the popular DESs reline and ethaline (1:2 ChCl:ethylene glycol), under similar synthetic temperatures (100–180 °C) to those used in hydrothermal methods, which apply highly concentrated NaOH solutions and cerium nitrate as the precursor^11^. Following reaction at 100 °C under autogenous pressure, the reline system gelled to a clouded rose-pink suspension, which could be precipitated using water as an anti-solvent. In contrast, the counterpart ethaline system underwent no apparent reaction; optical clarity was retained after the same treatment and the system was stable to the addition of anti-solvent H_2_O and ethanol with no solid precipitation even after several months. Consequently, the rest of the study was focused on the reline-based ceria synthesis method. Interestingly, it was found that the morphology of the resulting ceria materials could be finely tuned at the nanoscale by addition of controlled amounts of water. Several DES hydration ratios (*w*), defined as the H_2_O:reline molar ratio equal to 0, 2, 5 and 10, respectively, were used systematically. Water addition has the effect of diluting the DES, whereas remaining in a regime of hydration in which the DES structure is purportedly retained (that is, below 50 wt.% H_2_O; *x*_water_=0.83; *w*=14.4)^17^. The different ceria materials are accordingly labelled Ce-*x*-*y*, where *x* is synthesis temperature and *y* the DES molar hydration ratio (*w*). Representative transmission electron microscopic images of the synthesized ceria at different synthetic temperatures and water contents are shown in Fig. 1a. In general, syntheses from reline–aqueous mixtures yielded a white solid precipitate compared with a sol-gel in the pure reline synthesis, likely to be due to the formation of a nanoparticulate cerium compound network in the more viscous pure reline. Indeed, in the absence of added water (beyond the water of hydration found in the cerium nitrate precursor), only particulated ceria is formed, independent of the temperature of synthesis (100–180 °C). Increasing the water content (*w*) at constant synthesis temperature increases the aspect ratio of the materials, such that Ce-100-10 mainly comprise 1D nanowires of up to 10 nm diameter and several micrometres in length, revealing the role of water as directing agent due to the presence of hydroxyl groups, which have previously been shown to limit lateral growth^29^. In addition, as the synthesis temperature increases at loading DES hydration ratio of 10, the thickness and length of the 1D ceria structures increases, forming bundles. This route provides a green, non-toxic and biodegradable route for the synthesis of 1D ceria structures at mild conditions. It is fair to mention that milder conditions have been previously reported in aqueous solutions; however, very high (and corrosive) concentrations of base are required not only to direct the 1D growth but also to dissolve the cerium precursors^29^. To the best of our knowledge, this is one of the less intensive synthetic routes at which 1D ceria structures of these dimensions have been achieved, making this DES-solvothermal method a particularly mild set of conditions, highly attractive for the manufacturing of this type of nanomaterials using green, non-toxic and biodegradable solvents, with a 100% cerium yield.

### Solvothermal reaction mechanism

**Figure 2:**
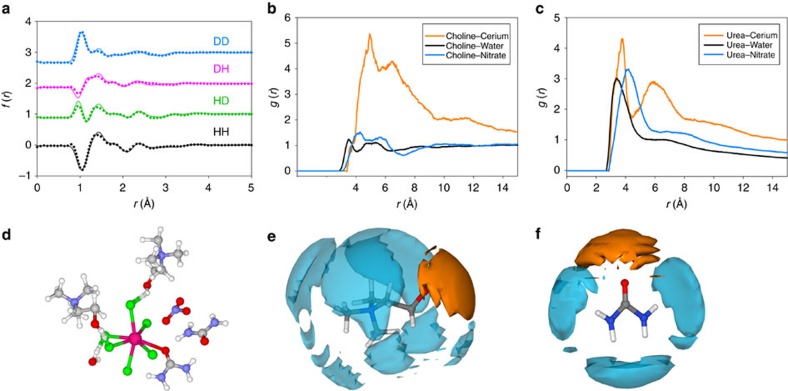
Results from neutron diffraction and EPSR analysis. (**a**) Experimental data (dotted lines) and EPSR fits (solid lines) for the four different reline isotopic contrasts. (**b**) Radial distribution functions (RDFs) of Ce(NO_3_)_3_.6H_2_O components around choline ions. (**c**) RDFs of Ce(NO_3_)_3_.6H_2_O components around urea molecules. (**d**) Snapshot demonstrating the variety in bonding interactions around the cerium ion taken from an iteration of the EPSR simulation. Bound chloride anions are stabilized by hydrogen bonding with choline and urea molecules, DES components ligate cerium via oxygen lone pair donation, and water and nitrate also contribute to the hydrogen bonding network. (**e**) Spatial density function (SDF) plot showing the 7.5% most probable 3D configurations of nitrate (blue) and cerium (orange) around choline ions. (**f**) SDF plot showing the 7.5% most probable 3D configurations of nitrate (blue) and cerium (orange) ions around urea molecules.

To determine the structure of the solvated cerium species in reline that underpins the mechanism of the solvothermal reaction, wide *Q*-range liquid-phase neutron diffraction was performed on pure reline–cerium nitrate mixtures at the synthetic cerium concentration and varying deuteration as isotopic contrast. Details of the computational methodology are provided in Supplementary Fig. 1 and Table 1, and the Supplementary Methods. We have recently demonstrated the structure of bulk reline using this method, showing hydrogen-bonded clusters of the eutectic stoichiometry, where a chloride ion is chelated by one choline and two urea molecules^30^. The calculated intermolecular coordination numbers for this system are shown in Table 1. The reline structure is tolerant to the addition of cerium nitrate hexahydrate, with around a 10% decrease in urea–urea coordination and a 10% increase in the choline–urea coordination numbers. Significant short range association of cerium and nitrate ions are observed around both choline and urea (Fig. 2). Both urea and choline participate in hydrogen bonding with nitrate anions, forming ordered clusters similar to the chloride cage that is formed in the pure DES. It is also interesting to note that the cerium nitrate water of hydration has a higher affinity for association with urea than choline via its hydrophilic functional groups and there is only vague cerium–water structuring at distances >5 Å. The cerium ion preferentially binds with four chloride anions and lone pairs of electronegative oxygen atoms, forming a near-charge balanced, highly fluxional complex that can be nominally described as [Ce^3+^(Cl)^−^_3.9_(Choline)^+^_1.1_(NO_3_)^−^_0.5_(Urea)_1.7_]^−0.3^. The strong Lewis acid–base interactions observed between Ce^3+^ and the ligating O atoms resemble a dilute form of the solvation structure of solvate ILs, which are eutectic mixtures of glymes and lithium salts^31^. Urea and choline molecules, as well as water molecules from the cerium nitrate hexahydrate, are capable of forming Ce–O bonds and simultaneously hydrogen bonding with ligated chlorides, allowing the integration of the complex ion into the greater hydrogen-bonding solvent structure without significant perturbation, as illustrated in Fig. 2d. Importantly, we found that the solvent structure drives reactive elements towards being tightly bound; cerium is ligated by urea, which itself forms a strong hydrogen-bonding network with water. It is likely to be that this facilitates both the hydrolysis of urea (shown in reaction (1)) and the targeted delivery of urea hydrolysis products (such as CO_3_^−^ and NH_4_^+^) towards these reactive cerium centres, with the solvent pre-structuring (as shown in reaction (2)) effectively reducing the reaction activation energy and therefore rationalizing the milder conditions of nanorod formation and growth.


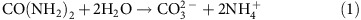



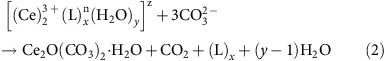


where (L)_*x*_^n^ describes the ligands solvating cerium, which can be charge-positive (choline), negative (chloride) or neutral (urea).

Further insights into the relationship between the nature of the DES and product morphology are required to elucidate the role of potential electrostatic interactions on capping surface lattice planes, similar to the ones observed in the presence of surfactants^32^, in promoting the 1D growth. It is important to mention the difference in temperature of the wide *Q*-range neutron-scattering experiments carried out at 303 K and actual ceria synthesis carried out under solvothermal conditions at the temperatures stated. However, the pre-ordering observed will occur in the reaction mixtures on mixing as they are loaded into the reactor and thus will form a starting configuration for the synthesis.

In contrast, in the ethaline DES synthesis, and although this remains to be tested in detail, we suggest that the Ce^3+^ ion is strongly chelated by non-hydrolysable ethylene glycol molecules in a similar manner, explaining the lack of product formation in this system. We therefore show here that the benefits of using reline as a solvent in ceria synthesis are afforded by the solvent playing the role of a latent supramolecular catalyst (as shown by reaction (2)), where the increase in reaction rate from solvent-driven pre-organization of the reactants is most significant.

Following calcination, X-ray diffraction (XRD) patterns of the nanoparticles synthesized from reline were fitted to the cubic ceria fluorite structure (JCPDS 34-0394). Pre-calcination structures synthesized in reline–water mixtures were assigned to orthorhombic monohydrated cerium oxycarbonate (Ce_2_O(CO_3_)_2_·H_2_O (JCPDS 44-0617)^33^, calcining to form ceria (Fig. 1b) as has previously been reported for urea-hydrothermal synthesis^34^ and concordant with the mechanisms predicted by our neutron diffraction analysis. In this study, samples were calcined at 500 °C to ensure full solvent removal but we have also demonstrated that conversion of cerium oxycarbonate to ceria takes place at temperatures as low as 200 °C, in line with earlier reports^34^. However, samples synthesized in the absence of added water (for example, Ce-100-0; Fig. 1b) showed characteristic diffuse ceria diffraction peaks before being calcined to remove amorphous reline remnants; nitrate anions held proximal to the cerium complex during synthesis in the pure reline environment may be capable of directly oxidizing it to ceria when conditions do not favour the urea-hydrolytic pathway, although interestingly in these cases 1D growth is not achieved. Alternatively, oxygen dissolved in these solutions can be responsible for such oxidation similar to previous observation in hydrothermal synthesis^29^. The thermal hydrolysis of urea above 80 °C to form CO_3_^−^ and NH_4_^+^ therefore occurs in both reline and reline–aqueous mixtures, as in aqueous urea solutions. We have found that the rate of this process is clearly suppressed at lower water concentrations and temperatures, with the reaction not running to completion after 10 h of solvothermal treatment at 80 °C. The underlying mechanism of crystal growth is therefore not the unique facet of the DES-based synthesis; carbonate ions react with solvated cerium centres to form oxycarbonates that grow into crystallites via an aggregation-dissolution-recrystallization-type mechanism^35^. The mean crystallite size (shown in Fig. 1c) of the different Ce-*x*-*y* materials ranges between 5 and 8 nm, with the differences within the experimental error, with the exception of the Ce-180-2 and Ce-180-5 materials, which show particularly large crystallites. However, 1D growth is still not achieved in this case, despite the apparent increase in aspect ratio observed for Ce-140-*y* materials relative to Ce-100-*y* materials, which is likely to be due to a greater degree of crystallite concatenation from more favourable growth kinetics as temperature is further increased to 180 °C.

### Porosity of the materials

**Figure 3:**
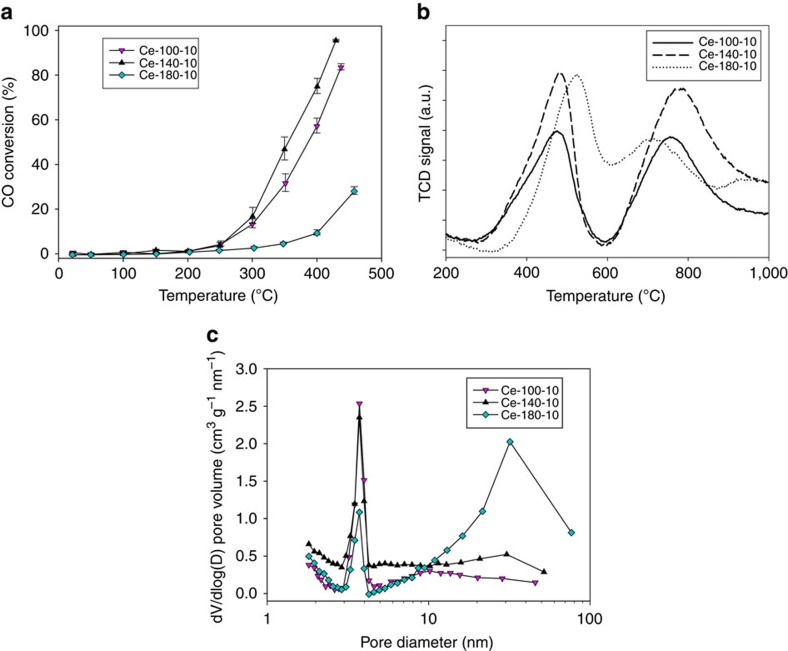
Structure–property relationships for the Ce-*x*-10 materials. (**a**) Catalytic CO oxidation conversion as a function of temperature (error bars represent the experimental error associated to the gas analyses used for the calculation of conversion values). (**b**) TPR (data normalized to mass of material). (**c**) Pore size distributions.

Although meso- and microporosity is desirable for ceria materials, the former often has poor thermal stability due to pore collapse, especially during calcination when surfactants are used in the synthesis^36^ and few microporous ceria synthetic strategies have been reported^37^. Herein, we find that ceria porosity can be tuned by careful selection of solvothermal reaction conditions, with porosity retained after calcination. As an example, Fig. 1d shows the N_2_ adsorption isotherms of the ceria nanorods formed in more highly hydrated (*w*=10) aqueous reline mixtures and the associated pore size distributions are shown in Fig. 3c. Despite presenting similar 1D morphologies, increasing the synthesis temperature favours the formation of larger mesopores and even macropores, with the former more likely to be due to the agglomeration of wires and rods. This relative loss of small pores can be attributed to more rapid aggregation and growth of crystallites as the dissolution/recrystallization kinetics scale with temperature. In addition, increasing the value of *w* favours the formation of microporous ceria. These effects can be applied synergistically to produce ceria materials with the desired porosity, in a solvothermal process, which is considerably less intensive than previously reported hydrothermal methods using highly concentrated base^29^,^38^.

### Carbon monoxide oxidation performance

In general, the CO oxidation reaction rate of reline-synthesized ceria products showed a comparable morphology relation to other ceria syntheses, with particularly high specific turnover frequency values found for ceria nanorods as compared with nanoparticulate materials, as these morphologies preferentially expose the more reactive surface lattice planes (100) and (110)^11^. Evaluation of the relationship between the synthesis conditions of the ceria materials and their physicochemical properties was carried out for the Ce-*x*-10 materials, all of which have a pure 1D morphology (Fig. 3). Further microstructure characterization of the different materials is shown in Supplementary Fig. 2 and Supplementary Table 2. Increasing synthetic temperature promotes the dissolution/recrystallization growth step (as previously observed with other 1D materials), leading to a decrease of the aspect ratio^39^. Ce-140-10 ceria nanorods, in particular rich in micro- and mesoporosity, are especially effective oxidation catalysts and competitive with the best literature examples of undoped ceria under comparable CO oxidation conditions with a rate of reaction of 69 μmol g^−1^ s^−1^ at 300 °C^29^. This high activity is directly related to the high reducibility of the high concentration of readily available surface oxygen as shown by the low temperature peak in the temperature-programmed reduction (TPR) analysis (Fig. 3b)^40^. The CO oxidation reaction is well known to follow the Mars van Krevelen mechanism where the adsorbed carbon monoxide reactant is oxidized by readily available surface oxygen species, creating an oxygen vacancy on the surface of the ceria material that is then restored by oxygen in the gas phase, closing the catalytic cycle. It is important to mention that bulk oxygen (whose concentration is proportional to the high-temperature peak in the TPR experiment) does not play a significant role in the catalytic activity of the material. Ce-100-10 ceria rods show a slightly lower oxidation activity than their Ce-140-10 counterpart due to a lower concentration of readily available oxygen species as shown by the decrease in intensity of the low-temperature peak in the TPR analyses (Fig. 3b)^40^, combined with relatively smaller mesopores (∼10 nm in Ce-100-10 versus ∼30 nm in Ce-140-10). On the other hand, the Ce-180-10 ceria material shows considerably lower oxidation catalytic activity despite comparable morphology, surface area (∼73–80 m^2^ g^−1^) and crystallite size (∼4–8 nm) with the other two materials. In this case, the ceria rods align into bundles with significantly higher mesopore volume. The accelerated kinetics of growth at this higher temperature is also reflected in the reducibility of the readily available oxygen at higher temperature, which is translated into a lower oxidation catalytic activity. Interestingly, reduction of the bulk oxygen (second peak of the TPR, Fig. 3b) of the Ce-180-10 ceria material takes place at relatively lower temperatures than the other Ce-*x*-10 materials; however, these species are not involved in the oxidation cycle. All samples were found to have stable performance over six consecutive temperature cycles between ambient temperature and 500 °C.

## Discussion

The reline system offers a feasible route for the synthesis of 1D ceria materials due to two complementary effects. On one side, the controlled degradation of the DES components, in this case urea, reacts with the cerium precursor salts while simultaneously the strong urea interaction with the metal ion facilitates the reaction path as demonstrated here. These aspects justified the lack of conversion observed with the ethaline system, which shows a strong interaction between the glycol and the metal ion but which does not undergo degradation to allow the reaction.

It is noteworthy that we attempted further structuring of the pure reline syntheses by adding the anionic surfactant SDS as a templating agent (3.8 wt.%; Ce:SDS molar ratio=0.33). This concentration of SDS in reline is known to form cylindrical micelles rather than the spheroids that are formed in water^41^. However, SDS added to the reline solvent did not act as organic templates during the ceria synthesis and only resulted in the formation of bulk ceria with negligible (10–12 m^2^ g^−1^) surface area and catalytic activity. Increasing the synthesis temperature (180 °C) caused surfactant pyrolysis. This lack of templating effect is likely to be related to SDS degradation and to the competing interactions between the SDS and the reline components with the cerium ions and the water molecules.

We have therefore demonstrated significant control over the morphology, porosity, reducibility and, consequently, the catalytic activity of nanostructured ceria materials by direct modification of the solvent environment and synthetic conditions. Current methods for synthesizing 1D ceria nanoparticles require either higher temperatures or longer synthesis times than the relatively mild conditions of this solvothermal method, although a post-calcination step is required to produce the final ceria product. Using DESs to synthesize ceria is therefore a step forward in both environmental and economic terms; this relatively mild route, using cheap, benign and biodegradable precursors alongside water as a structure-modulating agent, yields thermally stable ceria nanostructures (rods and particles) with equivalent activity to similar materials synthesized under conventional, more intensive conditions. Using liquid-phase neutron diffraction, we have demonstrated that cerium nitrate is readily integrated into the DES matrix of reline by hydrogen bonding and the formation of fluxional, chelated cerium complexes with the DES components, analogous to solvate ILs. Reline is not denatured by the addition of the cerium precursor salt and instead forms a pre-structured liquid where the reactive components are sandwiched together, with the solvent environment functioning as a supramolecular catalyst. This fundamental understanding sets the foundations of a new synthetic route, where the solvothermal method may also be used to directly produce ceria at even lower temperatures via ligated nitrate anions, potentially de-intensifying the process further by negating the requirement for calcination.

## Methods

### DESs and neutron diffraction

Reline was prepared as per the literature method^42^, from ChCl (≥98%) and urea (≥99.5%), used as received from Sigma-Aldrich. The water content of the prepared pure reline was found to be 2,252±519 p.p.m., using a Mettler-Toledo DL32 Karl Fischer titrator. Eutectic mixtures were prepared by mixing reline with Ce(NO_3_)_3_.6H_2_O (Acros, ≥99.5%). Neutron diffraction experiments used the SANDALS wide *Q*-range neutron diffractometer at the ISIS facility, RAL, UK^43^, using the previously described method, with 1:2:0.01 ChCl:urea:Ce(NO_3_)_3_.6H_2_O eutectic mixtures (equivalent to ∼43 mM Ce(NO_3_)_3_.6H_2_O) and ChCl:urea isotope-substituted contrasts of H:H, H:D, D:H and D:D^30^. Further background on the neutron diffraction technique can be found alongside the computational methodology in the Supplementary Methods.

### Deep eutectic-solvothermal synthesis of ceria

In a typical synthesis, 80 cm^3^ of either reline or reline and water (Elga, 18.2 MΩ) in the required molar ratio was added to a polytetrafluoroethylene (PTFE) autoclave liner. Ce(NO_3_)_3_.6H_2_O (1.5 g) was added to the vessel and homogenized before sealing. When specified, the surfactant SDS (Sigma-Aldrich, ≥99%) was added to the mixture at a Ce:SDS molar ratio of 0.33. The liner was placed inside a stainless steel autoclave, which was heated to the chosen temperature at 10 °C min^−1^ in an air-circulating oven, and held at this temperature for 10 h before cooling to ambient. The reaction mixture was diluted to 300 cm^3^ with water and centrifuged for 20 min at 7,500 r.p.m. The solids were separated by filtration, washed with water and ethanol, dried in a vacuum oven at 80 °C and calcined at 500 °C for 4 h with a ramp rate of 10 °C min^−1^. Grinding with pestle and mortar yielded a series of pale yellow powders of varying hues and densities, with a mean yield of 0.6 g.

### Characterization of nanostructured ceria

Powder X-ray diffraction used a Bruker D8 ADVANCE, equipped with a Bruker VÅNTEC-1 detector and Cu Kα radiation (*λ*=1.5418 Å). Average crystallite size is calculated applying the Scherrer equation to the diffraction peaks (111). The values corresponding to the other reflections are shown in the Supplementay Table 1. N_2_ sorption analysis was conducted using a Micromeritics ASAP 2020 at 77 K, after degassing under vacuum at 150 °C for 2 h. TPR measurements were performed using a Micromeritics ASAP 2920 instrument equipped with a thermoconductivity detector. The samples were treated under 50 cm^3^ min^−1^ of 5% H_2_/He gas, whereas the temperature was increased from ambient to 1,000 °C at 10 °C min^−1^. Imaging was performed using a JEOL JEM1200EXII (transmission electron microscope) and a JEOL FESEM6301F (field-emission scanning electron microscope) with no conductive coating and 2 kV accelerating potential. CO oxidation catalytic tests were carried out in a differential reactor inside a PID temperature-controlled oven. Ceria material (15 mg) were diluted in SiC to form a 4 cm^3^ catalytic bed. The gas inlet feed consisted of 50 cm^3^ min^−1^ of a mixture of CO/O_2_/N_2_ in molar ratio 0.2/0.2/99.6. Experiments were conducted from 25 to 500 °C with temperature increments of 50 °C. After reaching the steady state at each temperature, the outlet gas CO concentration was determined by a Fuji Electric ZRH Infrared Gas Analyzer.

### Data availability

The corrected wide *Q*-range liquid-phase neutron diffraction data are available via the Cambridge Repository system ( http://dx.doi.org/10.17863/CAM.4906). Raw data can be accessed via the ISIS-ICAT system (RB1510465). All other experimental data are available from the authors upon request.

## Additional information

**How to cite this article:** Hammond, O. S. *et al*. Deep eutectic-solvothermal synthesis of nanostructured ceria. *Nat. Commun.* **8,** 14150 doi: 10.1038/ncomms14150 (2017).

**Publisher's note**: Springer Nature remains neutral with regard to jurisdictional claims in published maps and institutional affiliations.

## Supplementary Material

Supplementary Information Supplementary Figures, Supplementary Tables, Supplementary Methods and Supplementary References

## Figures and Tables

**Table 1:** Intermolecular coordination numbers determined by EPSR.

**Molecule A**	**Molecule B**	***r***_**max**_ **(Å)**	***N***_**coord**_
Choline	Choline	8.1	6.33±1.89
Choline	Chloride	7.0	4.15±1.33
Choline	Urea	6.9	6.69±2.46
Urea	Choline	5.0	0.78±0.85
Urea	Chloride	5.5	2.14±1.02
Urea	Urea	6.2	6.11±2.53
Cerium	Choline	5.5	1.09±0.74
Cerium	Chloride	3.5	3.90±0.99
Cerium	Urea	4.3	1.67±1.11
Cerium	Nitrate	4.2	0.48±0.50
Water	Choline	4.0	0.37±0.54
Water	Chloride	4.4	0.85±0.57
Water	Urea	4.5	2.55±1.55
Nitrate	Choline	4.6	1.50±0.95
Nitrate	Chloride	5.5	1.60±1.04
Nitrate	Urea	5.5	3.50±1.56

EPSR, empirical potential structure refinement; RDF, radial distribution function.

The molecular centres for polyatomic species are taken to be the choline C_2_N atom, the urea CU atom, the O_1_ atom of water and the NN atom of nitrate. Intermolecular coordination numbers are determined using the first minima in RDFs (±0.05 Å) between molecules as the maximum radius of integration (*r*_max_) and the fluctuation in this value is calculated by EPSR over 6,000 iterations of the model.
