# Supplementary material for: Deep eutectic-solvothermal synthesis of nanostructured ceria
Source: Nat Commun. 2017 Jan 25;8:14150. doi: 10.1038/ncomms14150 (PMC5288492; doi:10.1038/ncomms14150)
Supplement: Supplementary Information — Supplementary Figures, Supplementary Tables, Supplementary Methods and Supplementary References [file ncomms14150-s1.pdf]

Supplementary Figures

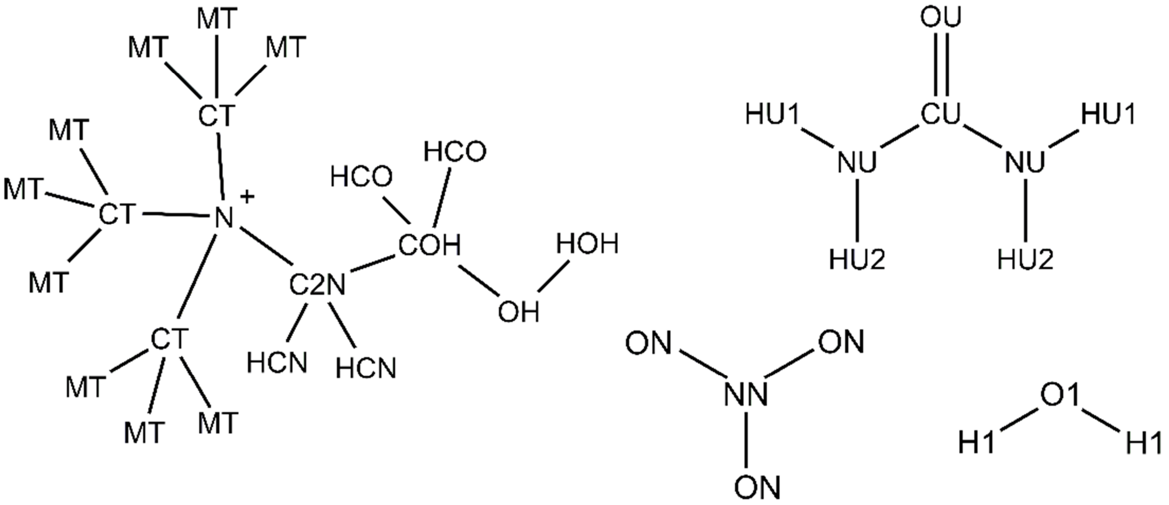

Supplementary Figure 1. Atom types and molecules used in the EPSR simulation.

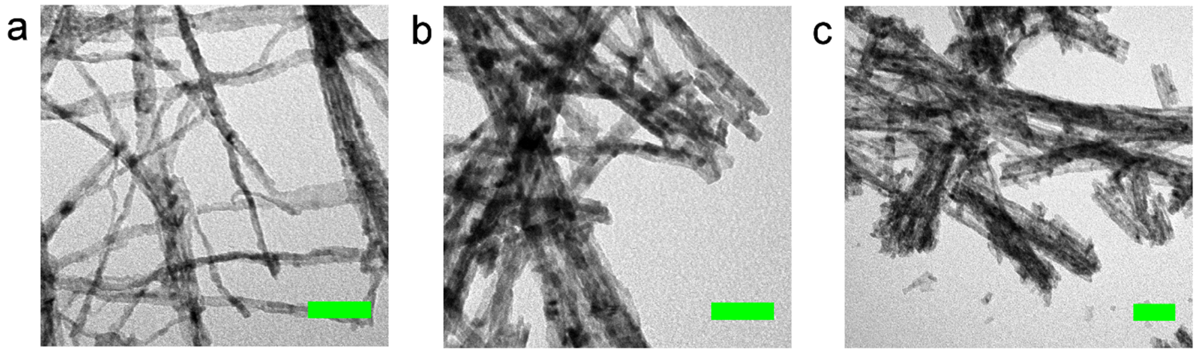

Supplementary Figure 2. Characteristic TEM micrographs of the Ce-y-10 materials. (a) Ce-100-10; (b) Ce-140-10; (c) Ce-180-10 (scale bars denote 50 nm). The three materials present a 1D morphology with high morphological purity. It is known that rods preferentially expose more reactive surface lattice planes (100) and (110). Increasing synthetic temperature promotes the dissolution/recrystallization growth step (as previously observed with other 1D materials) leading to a decrease of the aspect ratio.

Supplementary Tables

Supplementary Table 1. Assigned EPSR parameters for modelling of experimental diffraction data.

Atom type	ε (kJ mol^{-1})	σ (Å)	*q* (e)
N	0.700	3.200	1.000
C2N	0.800	3.700	-0.120
CT	0.800	3.700	-0.180
HCN	0.200	2.580	0.060
COH	0.800	3.700	0.145
MT	0.200	2.580	0.060
HCO	0.200	2.580	0.060
OH	0.650	3.100	-0.683
HOH	0.000	0.000	0.418
Cl	0.566	4.191	-1.000
CU	0.439	3.750	0.142
OU	0.878	2.960	-0.390
NU	0.711	3.250	-0.542
HU2	0.000	0.000	0.330
HU1	0.000	0.000	0.330
Ce	0.811	3.552	3.000
NN	0.711	3.250	0.845
ON	0.879	0.296	-0.615
O1	0.634	3.151	-0.834
H1	0.000	0.000	0.417

Supplementary Table 2. Scherrer crystallite size corresponding with XRD data.

	d / nm									
	{111}	{200}	{220}	{311}	{222}	{400}	{331}	{420}	{422}	Average
(2θ)	28.58	33.12	47.54	56.41	59.16	69.50	76.80	79.18	88.55	
Ce-180-0	8.40	4.97	7.73	8.16	6.67	6.62	7.17	5.76	5.71	6.80
Ce-140-0	7.72	4.89	7.07	7.29	6.73	6.65	6.73	5.89	5.07	6.45
Ce-100-0	8.33	5.93	8.38	9.29	11.64	11.93	8.94	9.27	8.28	9.11
Ce-180-10	7.05	6.65	5.96	6.80	2.21	4.57	5.29	3.56	3.36	5.05
Ce-140-10	8.82	8.99	6.92	6.67	5.60	4.97	4.55	5.02	3.57	6.12
Ce-100-10	9.09	7.17	7.66	7.79	9.84	11.73	7.28	7.40	6.48	8.27
Ce-180-2	24.74	17.66	25.76	25.57	22.44	21.45	18.70	19.30	15.71	21.26
Ce-140-2	6.39	4.06	5.45	5.83	4.90	4.95	5.51	3.99	4.15	5.02
Ce-100-2	7.45	6.44	6.97	7.16	8.83	7.41	6.16	4.90	4.98	6.70
Ce-180-5	17.36	13.16	18.69	19.32	15.09	17.59	14.84	14.62	12.47	15.90
Ce-140-5	7.85	7.22	8.32	7.65	8.92	9.50	6.25	6.95	5.97	7.63
Ce-100-5	8.16	5.21	6.91	7.48	10.14	11.24	7.43	6.83	6.15	7.73

The average crystallite size was determined by applying the Scherrer equation for all peaks that a Lorentzian function was fitted to, for the full angular range covered by the diffraction experiment (20-90 degrees 2theta). These diffraction peaks were: (111), (200), (220), (311), (222), (400), (331), (420), (422). The crystallite size calculated for each of the diffraction directions is similar to the average value, indicating the poly-crystallite nature of the 1D structures.

Supplementary Methods

Supplementary Method 1. Empirical Potential Structure Refinement Modelling Background. Empirical Potential Structure Refinement (EPSR) is a derivative of the Reverse Monte Carlo method, and simulates a 3D configuration that is objectively consistent with experimentally-determined diffraction data for a system.[1] This is achieved by using standard Lennard-Jones parameters and the known quantities of the system, such as molecular geometry and structure, density and composition, as well as permitting intramolecular disorder. The set of molecules used in the simulation are shown in Supplementary Figure 1, alongside their parameterization, in Supplementary Table 1. Parameters for urea are derived from previous diffraction experiments on high concentrations of aqueous urea,[2] and choline and chloride parameters hail from the OPLS All-Atom force field.[3] The bond lengths and molecular geometries of these species are as described previously.[4] Nitrate parameters are as used in analysis of diffraction experiments on alkylammonium nitrate ionic liquids,[5,6] and cerium parameters are derived from a 12-6-4 LJ-Type nonbonded model for highly charged metal centers.[7] Water is modelled using standard TIP3P parameters.[8]

Supplementary Method 2. Computational methodology. 215 choline, 215 chloride, 430 urea, 2 cerium, 6 nitrate and 12 water molecules are introduced to a simulation box which is then randomized. The initial density is set to 5% of the experimental value to allow reliable energy minimization. The model is allowed to run for a number of MC cycles until it has equilibrated in energy, whilst being compressed by approximately 10% per cycle until stabilizing at the experimental density.[9] The empirical potential is then introduced to refine the model to the neutron diffraction data, and approximately 6000 refinement cycles are performed to accumulate statistics on the structural information including intermolecular coordination numbers, radial distribution functions (RDFs) and spatial density functions (SDFs).

Supplementary Method 3. Intermolecular coordination number calculations. The COORD routine of EPSR runs alongside the fitting procedure, accumulating information on the intermolecular coordination numbers between different components of the system as the disordered system is allowed to evolve through the 6000 refinement cycles. Mean coordination numbers are therefore calculated alongside their respective errors, and the magnitude of these errors demonstrates the degree of intermolecular disorder between species. This calculation uses the true molecular center of mass for monoatomic species, but for polyatomic molecules the molecular centers are defined as the choline C2N atom, the urea CU atom, the O1 atom of water and the NN atom of nitrate. The resultant coordination numbers are shown in Table 1 in the main body text. Compared to the structure of the pure reline solvent, the structural change in the DES in terms of the intermolecular coordination can be seen to be minimal.[4]

Supplementary References

1. Soper, A. K. Empirical potential Monte Carlo simulation of fluid structure. *Chem. Phys.* **202,** 295–306 (1996).
2. Soper, A. K., Castner, E. W. & Luzar, A. Impact of urea on water structure: a clue to its properties as a denaturant? *Biophys. Chem.* **105,** 649–666 (2003).
3. Jorgensen, W. L., Maxwell, D. S. & Tirado-Rives, J. Development and Testing of the OPLS All-Atom Force Field on Conformational Energetics and Properties of Organic Liquids. *J. Am. Chem. Soc.* **118,** 11225–11236 (1996).
4. Hammond, O. S., Bowron, D. T. & Edler, K. J. Liquid structure of the choline chloride-urea deep eutectic solvent (reline) from neutron diffraction and atomistic modelling. *Green Chem.* **18,** 2736–2744 (2016).
5. Hayes, R., Imberti, S., Warr, G. & Atkin, R. Amphiphilicity determines nanostructure in protic ionic liquids. *Phys. Chem. Chem. Phys.* **13,** 3237–3247 (2011).
6. Hayes, R., Imberti, S., Warr, G. & Atkin, R. Pronounced sponge-like nanostructure in propylammonium nitrate. *Phys. Chem. Chem. Phys.* **13,** 13544–13551 (2011).
7. Li, P., Song, L. F. & Merz, K. M. Parameterization of Highly Charged Metal Ions Using the 12-6-4 LJ-Type Nonbonded Model in Explicit Water. *J. P* **119,** 883–895 (2015).
8. Jorgensen, W. L. *et al.* Comparison of simple potential functions for simulating liquid water Comparison of simple potential functions for simulating liquid water. *J. Chem. Phys.* **79,** (1983).
9. Hargreaves, R., Bowron, D. T. & Edler, K. Atomistic structure of a micelle in solution determined by wide Q-range neutron diffraction. *J. Am. Chem. Soc.* **133,** 16524–16536 (2011).
